# Notes on the Treatment of Some Common Ocular Affections
*Communicated to the Bristol Medico-Chirurgical Society on 13th April, 1932.


**Published:** 1932

**Authors:** E. R. Chambers

**Affiliations:** Surgeon, Eye Hospital, and Ophthalmic Surgeon, Royal Infirmary, Bristol


### NOTES ON THE TREATMENT OF SOME COMMON OCULAR AFFECTIONS.*

BY

E. R. Chambers, F.R.C.S., D.O.M.S.,

*Surgeon, Eye Hospital, and Ophthalmic Surgeon, Royal Infirmary, Bristol.*

---

In this short paper, which is rather disjointed, I want to bring to your notice a few facts connected with common ocular diseases.

*Conjunctivitis.*—If we consider the pain caused by this condition, we find it is always of a gritty or burning type, and very often the patient is convinced that he has a foreign body in his eye, as this may produce exactly similar symptoms. The character of the pain is of great importance from a differential diagnosis point of view, as it at once excludes such conditions as iritis and subacute glaucoma. The inflammation, or vascular injection, is also typical, the blood vessels being brick-red, tortuous, and moving with the conjunctiva because they are in it. They are largest at their origin, which is at the periphery of the eye, and gradually diminish in size as they near the corneal margin. There is always a discharge in conjunctivitis, but in mild cases this is not obvious: its presence can often be ascertained by everting the lower lid, and letting the sac fill with tears, when beads of discharge will be seen floating in the clear tears, or

* Communicated to the Bristol Medico-Chirurgical Society on 13th April, 1932.

the patient may state that his lids are slightly stuck together when he wakes in the morning.

Now, as regards treatment, lotions and drops are all very well, but I am convinced that in order to cut an attack short there is no better treatment than painting the lids immediately the case is diagnosed, and it is the only efficient means for a case that has persisted in spite of treatment. One per cent. mercurochrome is as good as anything. Use a fine probe with a very little cotton wool, anæsthetise the eye with a drop of four per cent. cocaine, and in dealing with the upper lid do not evert it, but tell the patient to look down, catch hold of the lashes, and raise it from the eyeball, and then slip the probe under: by this method the painting is carried right to the top of the fornix, and so applied to the whole of the conjunctiva, whereas if the lid is everted and then painted the fornix is not touched.

Many cases of conjunctivitis will persist in spite of treatment, unless this includes nasal antiseptics. There need not necessarily be a history of nasal catarrh. The infection spreads to the eye via the nasal duct, and although antiseptics applied to the eye run down the duct into the nose, they are, apparently, not sufficient to deal with the infection.

*Iritis.*—The pain in this condition is very typical, and quite different from that of conjunctivitis. It is not in the eye itself, but in the bones of the orbit and face, and is of a neuralgic character. The vascular congestion is typical: it consists of straight, hair-like vessels of a lilac colour situated beneath the conjunctiva, and having their maximum intensity around the corneal margin. In iritis there is no discharge, but an increase in clear and normal tears. A considerable aid in the diagnosis of iritis is to compare the irides of the two eyes : the affected one will show a contracted pupil, and change in its normal colour, and the marking on its surface will be obscured by œdema. The vision is always considerably reduced in iritis.

In investigating the cause of a primary iritis every endeavour should be made to exclude any septic focus. As a result of complete pathological investigation made over many years, we have found the teeth, accessory nasal sinuses, and prostate mainly responsible. As regards the teeth, there is no doubt that the apical abscess is the most responsible, and as it occurs in the well-cared-for mouth, and can only be recognized by an X-ray examination, it is often missed. The prostate should always be suspected with recurrent iritis in the young male, as we have conclusively proved that the gonococcus can lie dormant there for many years. Since complete pathological investigations are now made in cases of iritis, it is interesting to note that the so-called "rheumatic iritis" is far less common than in former years.

I will not trouble you with the treatment of iritis, which is well known, but there are one or two points worth emphasizing. For instance, sodium salicylate is by far the best drug to give internally, but it must be given in large doses. Heat, applied in some form or other to the eye, should never be omitted. The thin, small pad of cotton wool worn under a shade should be discarded, and a thick piece of gamgee tissue extending well beyond the orbit is the only means of keeping the eye well protected and at an even temperature.

*Squint.*—I will deal with the non-paralytic or concomitant variety. A squint occurring in a child up to twelve months of age is of little importance unless it is considerable and fixed permanently in one eye. A child up to this age has acquired very little power of fixation, and in many cases, as this develops, the squint will disappear. After twelve months a squint should receive definite attention, as it is not likely to recover spontaneously, and will probably get worse.

To be able to undertake the treatment of squint intelligently it is necessary to realize that we are endeavouring (1) to prevent the squinting eye from becoming blind from disuse, and (2) so to educate and train the faculty of fusion that the child uses both eyes, fuses the two retinal images, and thus has binocular vision. Now, in order to prevent the squinting eye from becoming blind it must be brought into use, and this can only be done by excluding all or some of the vision from the straight eye. The most efficient way to carry this out is to exclude the straight eye altogether by strapping a pad over it. This is probably the best treatment if the child is not too young, and if it possesses useful vision in the squinting eye. It is, however, impossible to carry this out in young children, as they will not keep the pad on, and some method has to be adopted which they cannot remove from the eye. Atropine is most useful in these cases, as it paralyses the accommodation, and so, at any rate for near objects, puts the eye out of action. An instillation of a 1 per cent. solution once a week is usually sufficient. Whether atropine or some other method of occlusion is adopted, it will often be found that after prolonged treatment the original squinting eye becomes straight, and the squint appears in the other. This is an advantage, as it is definite evidence that the treatment has achieved the required results, and that each eye has almost certainly good vision. In this way a squint may be made to alternate between the two eyes until the child is old enough for something more to be done. Never put atropine into both eyes at the same time.

As you know, squinting children practically always have some error of refraction, and it is of the greatest importance that this should be investigated. One is often asked at what age a child should be examined for and, if necessary, fitted with glasses. I would suggest that there is little harm in waiting until the age of two years, if in the meantime the atropine treatment is carried out. I know that some oculists attempt to make a child wear glasses whilst it is still in the cradle, but perhaps babies differ in different parts of the world.

Glasses having been prescribed, it is of the greatest importance that they should be worn constantly. Mothers often object to this, but the squint will not improve if the parents are allowed to have their way. Nor does the treatment of squint cease with the prescribing of glasses. The child may still be amblyopic in one eye, and the fusion faculty need training. In young children atropine may still have to be instilled into the straight eye, or in older children an occlusion disc worn over this eye to encourage the squinting eye to work. As the child becomes more intelligent every endeavour must be made to train the fusion faculty. This is best carried out by daily exercises with some form of stereoscope; five or ten minutes a day is quite sufficient for young children, and the child need not become bored with one set of pictures, as there are now so many different varieties made specially for this purpose of fusion training. It is interesting to note that as the training of squinting children has been better understood, and so more adequately carried out, the numbers requiring operation have greatly diminished.

*Foreign Bodies in the Eye.*—These are most commonly found on the surface of the cornea and on the conjunctiva of the upper lid. If you find a foreign body in the cornea never fail to evert the upper lid, and look for one in this position also ; it will often be found, and you will derive no credit for the removal of the obvious one if you leave the other. In removing a foreign body from the cornea use a sharp spud, and insert the point just under it and lift it out, your object being to remove as little of the epithelium as possible, as this is the chief barrier in preventing corneal infection. Blunt spuds must necessarily scrape off much of the epithelium. After the removal of a foreign body from the cornea some antiseptic should be inserted into the conjunctival sac, and this should preferably be an ointment, as its oily nature allays pain. We use an ointment of 1 per cent. mercurochrome or 1 per cent. iodoform. The eye, having been anæsthetized with cocaine, for the removal of the foreign body, remains insensitive to dust, etc., for some time; for this reason, and also in order to let the epithelium regenerate, a pad should always be worn over the eye for twenty-four hours.

*Blepharitis.*—This consists of an infection, usually staphylococcal in origin, and of the glands at the lid margin, and later of the hair follicles themselves. Although a very common ocular condition, it is one of the most neglected, yet it may often lead to much discomfort in after years. Those eyes which are very sensitive to such irritants as wind, dust, sun, etc., often owe their trouble to a neglected blepharitis; also such conditions as ingrowing eyelashes, with consequent corneal irritation, can be ascribed to the same condition. Recurrent mild attacks of conjunctivitis will often rapidly disappear if the infection of the lid margin is dealt with.

The object aimed at in the treatment of blepharitis is to clear the infection, not from the surface of the lid margin, but from the glands and gland ducts. This can often be efficiently carried out by the so-called method of milking the lid glands. After cocainizing the conjunctiva, the edges of the lids are held with the thumb and forefinger, and squeezed with a movement towards their edges: the effect of this treatment is often surprising in a case which has withstood other methods of treatment, and it should be more generally adopted. In blepharitis one often finds that some antiseptic ointment has been prescribed, with instructions that it be smeared over the edges of the lids. This is often useless, as thick and hard scabs have formed, so that the ointment never reaches the lid margins. The scabs must therefore be first removed, and this must be done with great care, so that the underlying granulation tissue is not injured, as, if it is, fibrous thickening of the lid margins may result, with the formation of entropion or ectropion. The best way to remove these scabs, and this should always be done before any ointment is applied, is to soak the lid margins gently with a warm solution of sodium bicarbonate, which is applied with cotton wool swabs.

In the case of children with blepharitis never fail to exclude the presence of infected tonsils and adenoids; it is surprising how many cases will clear up after the nose and throat have been attended to.

Blepharitis rarely occurs in a healthy child, and every endeavour should be made to improve the general condition. General ultra-violet light baths are of definite value, and a change to country air is beneficial, but sea air often does these cases more harm than good, unless protecting glasses are worn.

Autogenous vaccine treatment is very valuable, and in severe cases the vaccine should be injected under the skin, close to the lid margin.

In conclusion, just a few words about atropine. Never use it indiscriminately, but only when you are certain of your diagnosis. Remember that it is the most powerful pupil dilator we possess, and its action cannot be counteracted by any other drug. if there is any doubt, use homatropine, which can be counteracted with eserine. Atropine is a severe irritant to the conjunctiva if used over long periods, and acute glaucoma induced by atropine is far from being unknown.

